# Differential susceptibility of cariogenic and commensal oral *Streptococcus* species to chemically distinct disinfectants including ozone water *in vitro*

**DOI:** 10.1016/j.infpip.2026.100543

**Published:** 2026-04-15

**Authors:** M. Komatsubara, A. Watanabe, S. Kuwagi, J. Uchiyama, K. Gotou, K. Yokota

**Affiliations:** a Department of Bacteriology, Academic Field of Health Sciences, Okayama University, Okayama, Japan; b Oral Health Care Promotion, School of Oral Health and Welfare, Faculty of Dentistry, Tokushima University, Tokushima, Japan; c Department of Bacteriology, Graduate School of Medicine Dentistry and Pharmaceutical Sciences, Okayama University, Okayama, Japan

**Keywords:** Streptococcus mutans, Non-mutans streptococci, Biofilm, Ozone water

## Abstract

**Background:**

*Streptococcus mutans* is the primary causative agent of dental caries. Many *Streptococcus* species present in the oral cavity are thought to increase the risk of infective endocarditis and pneumonia. Conversely, *S. sanguinis*, *S. oralis*, *S. salivarius*, *S. gordonii*, and *S. mitis* are streptococci frequently isolated from the oral cavity and are thought to suppress the colonisation of cariogenic bacteria. Recently, mouthwashes have gained attention as a preventive strategy against dental caries. Therefore, we conducted an experiment to verify the caries prevention effect of various disinfectants on not only *S. mutans* but also other oral resident bacteria by comparing the number of surviving bacteria and the total amount of biofilm.

**Methods:**

The effectiveness of the disinfectants tested included two commercially available antiseptics with different mechanisms of action, as well as ozone water.

**Results:**

Crystal violet staining indicated that a 30-s exposure to these disinfectants was largely ineffective in removing biofilms. Adenosine triphosphate (ATP) assays revealed that *S. mutans* exhibited greater resistance to bactericidal action than non-cariogenic bacteria. Among the three disinfectants used in this experiment, ozone water had the lowest ATP value for *S. mutans* and showed no significant bactericidal effect against non-mutans Streptococci.

## Introduction

*Streptococcus mutans* is recognised as the primary causative agent of dental caries. However, certain members of the oral microbiota can inhibit the activity of cariogenic bacteria. For example, *Streptococcus mitis*, *Streptococcus sanguinis*, *Streptococcus oralis*, and *Streptococcus salivarius* are thought to inhibit the growth of *S. mutans* by producing hydrogen peroxide (H_2_O_2_). Similarly, *Streptococcus gordonii,* which is frequently isolated from the normal oral cavity, is believed to suppress the colonisation of cariogenic bacteria [1,2]. Therefore, when using mouthwashes to prevent dental caries, the ideal aim is not only to reduce the total bacterial load in the oral cavity but also to preserve the balance between cariogenic bacteria and those that inhibit their activity [3].

This oral flora generally colonises oral tissues, such as the mucosal epithelium, tooth surfaces, and gingival sulci, through the formation of biofilms. Biofilms are formed by microbial cells surrounded by an extracellular matrix, which enhances adhesion to solid surfaces and protects them from disinfectants [4,5]. In particular, *S. mutans* exhibits several traits that enhance its cariogenic potential during biofilm formation: (i) the ability to synthesise large amounts of glucan from sucrose to produce a water-insoluble extracellular matrix; (ii) the ability to metabolise various carbohydrates into organic acids; and (iii) the capacity to grow under environmental stress, especially in low-pH environments. These biofilm-forming capabilities are believed to contribute significantly to the high cariogenicity of *S. mutans* [6].

Ozone water is prepared by dissolving ozone in water. Like ozone gas, ozone water possesses strong oxidising power and is utilised for disinfection, sterilisation, decolourisation, and deodorisation [[7], [8], [9]]. Ozone water offers the advantages of being non-residual (half-life: 20–30 min) and causing minimal adverse effects on living organisms [10]. On this basis, ozone water has become widely used in Japan for cleaning and disinfecting dental equipment, gargling, drill cooling, and disinfection during implant surgery. Nevertheless, when used as an over-the-counter product, the non-residual property of ozone water is regarded as a disadvantage, as it cannot be stored for extended periods of time. In recent years, however, compact ozone water generators for domestic use have become readily available [11], and several products are now marketed specifically for gargling. Furthermore, a previous study in Japan evaluated the safety of ozone water by conducting acute oral toxicity tests in rats and primary as well as cumulative skin and eye irritation tests in rabbits using 7 ppm ozone water. The results indicated that 7 ppm ozone water exhibited none of the above toxicities [12].

Against this background, this study aimed to assess the effects of various disinfectants on total biofilm mass and bacterial survival across different oral streptococci. Specifically, we sought (i) to verify the caries-preventive potential of disinfectants while considering the preservation of the normal oral microbiota, and (ii) to evaluate the relative effectiveness of ozone water in caries prevention compared with other over-the-counter antiseptic products.

## Methods

### Strains used and various disinfectants

The strains used in this experiment were *S. mutans* (NBRC 13955), *S. mitis* (NCTC 3865), *S. sanguinis* (ATCC 10556), *S. oralis* (ATCC 10557), *S. gordonii* (ATCC 10558) and *S. salivarius* (ATCC 7073). These strains were cultured on 5% horse blood HI agar medium at 37°C in a 10% CO_2_ environment for 24 h.

Three types of disinfectants were used in the experiments: commercial antiseptic 1 (active ingredients: cetylpyridinium chloride, benzethonium chloride, and alcohol), commercial antiseptic 2 (active ingredient: povidone–iodine), and ozone water (4–5 mg/L). The ozone water was generated electrolytically from distilled water using the O3FIX® generator (ITRON Inc., Tokyo, Japan), and the dissolved ozone concentration of the generated ozone water concentration was measured using the DOC-O5A® ozone checker (Ebara Jitsugyo Co., Ltd, Tokyo, Japan) before every experiment.

### Measurement of total biofilm volume and viable bacterial count

#### Culture conditions and disinfectant action conditions

One millilitre of Trypticase Soy Broth (TSB) medium containing 1% sucrose and 0.2% yeast extract (hereafter referred to as ‘TSB medium’) was dispensed into each well of a 24-well microplate, and the above *Streptococcus* spp. were inoculated into three wells and then incubated statically for 24 h at 37°C in a 10% CO_2_ environment. After incubation, the TSB medium was removed and the wells were washed once with 1 mL of phosphate-buffered saline (PBS). Subsequently, 1 mL of distilled water or various disinfectants (commercial antiseptic 1, commercial antiseptic 2, or ozone water) was dispensed into each well and allowed to act for 30 s. The wells were washed once with 1 mL of PBS.

#### Quantification of viable bacteria by ATP measurement

To each well of the samples prepared above, 500 μL of saline was added, and the biofilms were resuspended by pipetting 10–20 times. The entire biofilm mixture from each well was then transferred to a 1.5-mL microcentrifuge tube (Eppendorf, Hamburg, Germany). Adenosine triphosphate (ATP) levels of the biofilm suspension in each microcentrifuge tube was then measured using a Lumitester PD-20 (Kikkoman Biochemifa Corporation) and LuciPac A3 Water (Kikkoman Biochemifa Corporation).

### Observation of stained images using a confocal laser scanning microscope

#### Culture conditions and disinfectant action conditions

Each well of a CELLview® Glass Bottom Dish, 35 mm, four compartments, tissue culture-treated (Greiner Bio-One, Kremsmünster, Austria), was filled with 600 μL of TSB medium and inoculated with Streptococcus spp. Cultures were incubated statically for 24 h at 37°C in a 10% CO_2_ atmosphere. Following incubation, the TSB medium was removed, and the wells were washed once with 600 μL of PBS. Subsequently, the wells were treated with 600 μL of distilled water or one of the disinfectants (commercial antiseptic 1, commercial antiseptic 2, or ozone water) for 30 s, followed by a single wash with 600 μL of PBS.

#### Diamidino-2-phenylindole staining

Each well of the samples prepared received 400 μL of diamidino-2-phenylindole (DAPI) solution diluted to 2 μg/mL in PBS. After 10 min of staining at room temperature in the dark, the stained images were observed using a ZEISS LSM780 confocal laser scanning microscope (Carl Zeiss Microscopy GmbH, Jena, Germany). A 40× objective lens was used to capture all samples, with excitation/emission filter wavelengths of 405/410–585 nm and a resolution of 1024 × 1024 pixels. Image data acquired under the above conditions were processed using ZEN 3.0 SR black edition software (Zeiss).

#### LIVE/DEAD biofilm staining

Biofilm staining was performed using the LIVE/DEAD® BacLight™ Bacterial Viability Kit L7012 (Thermo Fisher Scientific). An equal mixture of 3.34 mmol SYTO9 dye (Component A) and 20 mmol propidium iodide (Component B) was diluted with PBS to a final concentration 3 μL/mL, and 400 μL was added to each well of the samples. After 15 min of staining at room temperature in the dark, the stained images were observed using a ZEISS LSM780. All samples were photographed using a 40× objective lens, with excitation/emission filter wavelengths set to 488/493–560 nm (live; green) and 561/561–712 nm (dead; red), at a resolution of X × Y = 1024 × 1024. Image data obtained under the above conditions was processed using a ZEN 3.0 blue edition (Zeiss). Ratio of dead cells (dead cells/total cells) was also analysed by ZEN software.

### Statistical analysis

Experimental results of ATP assay are presented as mean ± standard error. Statistical analysis was performed using the Tukey–Kramer multiple comparison test, with *P*<0.05 considered statistically significant.

## Results

### Quantification of viable bacteria by ATP measurement

**Figure 1:**
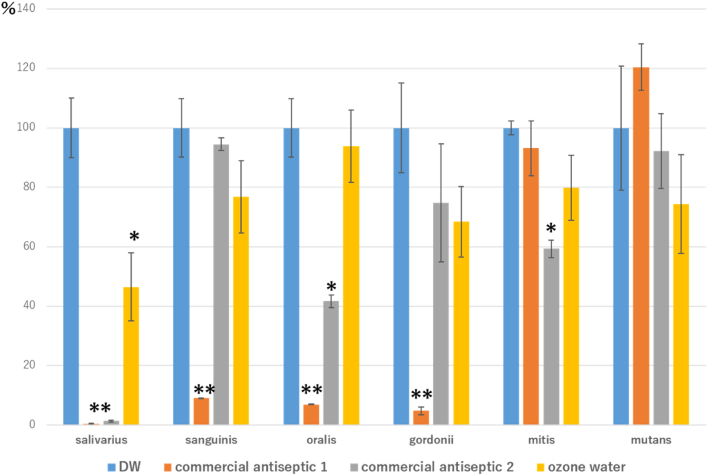
Adenosine triphosphate (ATP) quantification results (100% as distilled water (DW) exposure) for *Streptococcus* spp. after exposure to various disinfectants. Each graph shows the mean and standard error of *N* = 6. ∗ *P*<0.05 and ∗∗ *P*<0.01 vs each bacterium treated with DW, indicating a statistically significant difference.

Bacterial survival, assessed using ATP as an indicator, varied among species. Commercial antiseptic 1 effectively reduced ATP levels in *S. salivarius, S. sanguinis, S. oralis,* and *S. gordonii,* even within biofilms. Most disinfectants also decreased ATP levels in *S. salivarius, S. sanguinis, S. oralis, S. gordonii,* and *S. mitis.* In contrast, *S. mutans* exhibited no significant reduction in ATP levels with any of the disinfectants, and values remained high. Among the three disinfectants, ozone water was the most effective at reducing the number of viable *S. mutans* (Figure 1).

### Observation of stained biofilm images using a confocal laser scanning microscope

**Figure 2:**
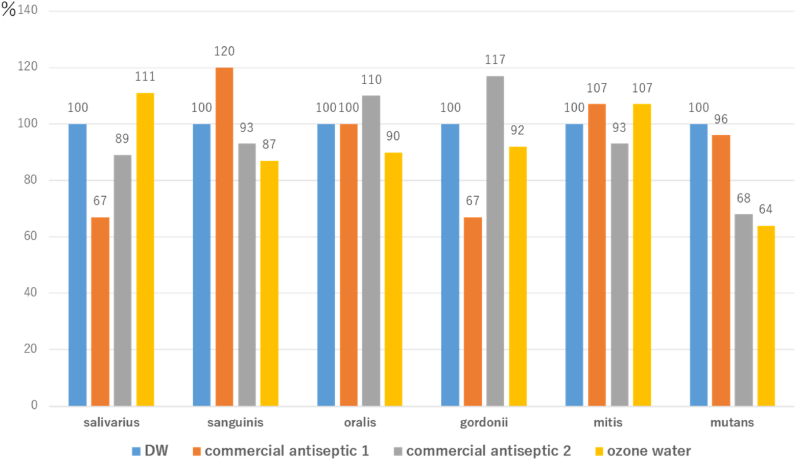
Maximum biofilm thickness (100% as distilled water (DW) exposure) of *Streptococcus* spp. after exposure to various disinfectants. The Z-value, representing the maximum biofilm thickness in the visualised area using ZEISS LSM780, is plotted as the Z-axis width.

In the DAPI-stained images, biofilm thickness (Z-axis width) was compared between control samples and those treated with distilled water or disinfectants (commercial antiseptic 1, commercial antiseptic 2, and ozone water). Control biofilms of *S. sanguinis, S. mutans* and *S. mitis* were thicker than those of the other three bacteria, indicating robust biofilm formation (Table I). *S. mitis* biofilms were reduced by treatment with distilled water and disinfectants. *S. mutans* maintained a thick structure in distilled water and commercial antiseptic 1, whereas biofilm formation was reduced by commercial antiseptic 2 and ozone water (Figure 2).

**Table I:** Maximum biofilm thickness (μm) of *Streptococcus* spp

	Salivarius	Sanguinis	Oralis	Gordonii	Mitis	Mutans
Distilled water	4.48	7.46	4.98	5.97	7.46	12.44
Commercial antiseptic 1	3	8.96	4.98	3.98	7.96	11.94
Commercial antiseptic 2	3.98	6.97	5.47	6.97	6.97	8.46
Ozone water	4.98	6.47	4.48	5.47	7.96	7.96

**Figure 3:**
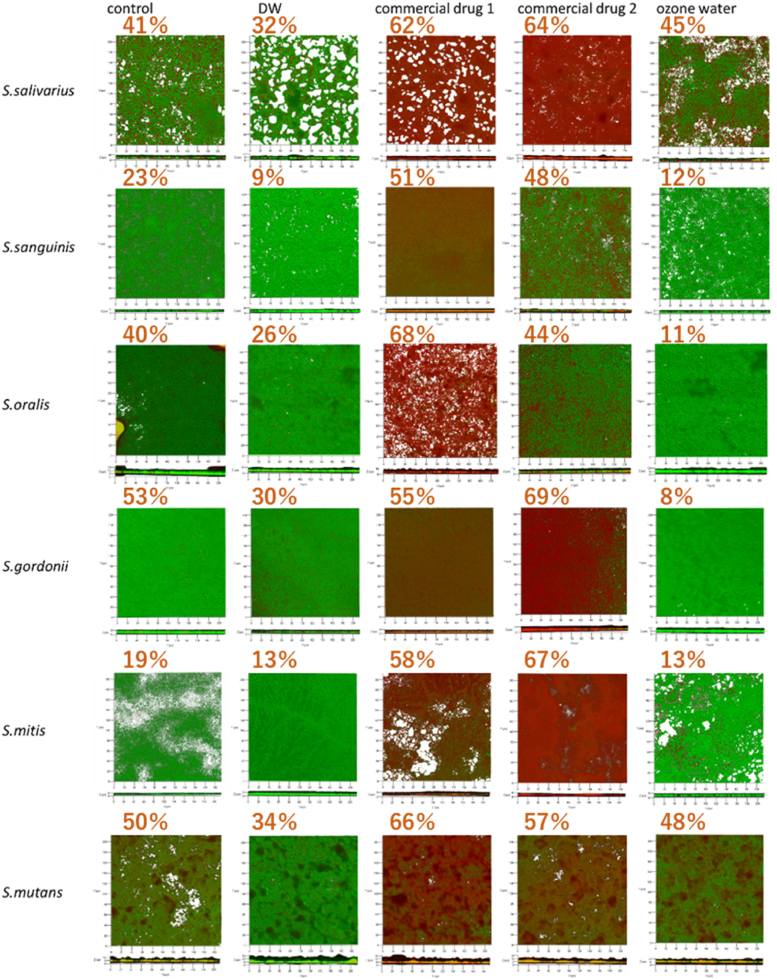
LIVE/DEAD-stained biofilm (LIVE = green, DEAD = red). Frontal three-dimensional images and cross-sectional images of the bottom edge of the frontal image were generated over an area of X = 200 μm × Y = 200 μm. Numbers were dead cells (%) analysed by ZEN software. DW, distilled water.

LIVE/DEAD biofilm staining was performed to evaluate SYTO9 (live bacteria, green) and PI (dead bacteria, red). Mixed images for ozone water showed a higher proportion of live bacteria compared with those treated with commercial antiseptics 1 and 2, indicating that ozone water had a lower bactericidal effect against non-*mutans* streptococci than the two commercial antiseptics used in this experiment. In contrast, the PI ratio for *S. mutans* in ozone water was higher than in the distilled water control, indicating that ozone water exerts a measurable bactericidal effect against *S. mutans* (Figure 3).

## Discussion

*S. mutans* is a key contributor to the development of dental caries [6], and effective oral care should aim to sterilise, remove, and inhibit its growth. Additionally, more than 700 types of resident bacteria are believed to coexist in the oral cavity, including species that do not cause dental caries [13], but can suppress the activity of cariogenic bacteria. Therefore, optimal oral care for caries prevention should not only reduce the total bacterial load in the oral cavity but also preserve the resident oral microbiota [[1], [2], [3]]. This study suggests that 4–5 ppm ozone water exerts a bactericidal effect against *S. mutans*, while preserving the resident oral microbiota.

Crystal violet staining is a commonly employed technique for quantifying biofilms in microplates, as it comprehensively stains live and dead cells as well as extracellular matrix components in biofilms, making it suitable for quantitative analysis of total biofilm biomass [8,14]. In this study, Crystal violet staining revealed no significant differences between the control and samples treated with distilled water or disinfectants (commercial antiseptic 1, commercial antiseptic 2, and ozone water) under any conditions, except for *S. salivarius* treated with commercial antiseptic 1. This suggests that a 30-s exposure to the tested disinfectants is generally insufficient to remove established biofilms.

An ATP-based quantitative assay, utilising the luminescence principle of the enzyme cycling method, was also employed in this study to estimate viable cell counts in biofilms of six *Streptococcus* spp. ATP is produced and maintained exclusively within living cells, and its concentration is proportional to the volume of living cells [14,15]. The results indicate that *S. mutans,* a cariogenic bacterium, is less susceptible to bactericidal action than non-cariogenic bacteria such as *S. mitis, S. sanguinis, S. oralis, S. salivarius*, and *S. gordonii.* Among the three disinfectants tested, ozone water reduced ATP levels in *S. mutans* most effectively, indicating no significant bactericidal effects on non-*mutans* streptococci. This suggests the potential for caries suppression while maintaining the balance of the normal oral microbiota.

A previous study examining the bactericidal effect of ozone water on *S. mutans* biofilms reported that 5 ppm ozone water, generated by a dielectric barrier discharge ozone generator, did not significantly reduce viable cell counts in *S. mutans* biofilms [16]. This result is consistent with the lack of a significant decrease in the ATP levels of *S. mutans* observed in this study. However, no previous research has comparatively assessed the bactericidal effect of ozone water against *S. mutans* and other oral commensal bacteria. This study therefore demonstrates that ozone water may exert a relatively higher bactericidal effect against *S. mutans* compared with other bacterial species, highlighting its potential value in caries prevention.

The bactericidal mechanism of commercial antiseptic 1 is electrostatic binding to the bacterial body, bactericidal action on bacteria on the surface of the biofilm, and inhibitory effect on biofilm growth. The bactericidal mechanism of commercial antiseptic 2 is oxidative bactericidal action by halogen, and penetration and sterilisation deep into the biofilm. Both disinfectants were effective against biofilms of all oral streptococci, including *S. mutans*.

The specific bactericidal effect of ozone water against *S. mutans* observed in this study is likely due to its oxidative mechanism, which exerts a stronger bactericidal effect on *S. mutans*, a highly anaerobic species, than on other *Streptococcus* spp. For example, bacterial species that initially adhere to tooth surfaces, such as *S. sanguinis*, efficiently metabolise glucose in the aerobic surface layer of dental plaque, whereas *S. mutans* rapidly produces acid in the highly anaerobic deeper layers [17]. Furthermore, the non-mutans streptococci used in this experiment produce H_2_O_2_, generating oxidative stress within oral biofilms and competing with H_2_O_2_-sensitive bacteria, whereas *S. mutans* is known to be sensitive to H_2_O_2_ [1,2,18]. However, this study used biofilms of *Streptococcus spp*. cultured *in vitro*, whereas the oral environment contains many elements that cannot be fully reproduced *in vitro*, such as salivary components (e.g., β2-microglobulin, lysozyme) and the complex coexistence of diverse resident bacteria [19,20]. Further investigations are therefore needed to determine whether these findings are applicable in the oral environment or under conditions that more closely resemble it.

Additionally, the three disinfectants used in this experiment were unable to completely remove the biofilms, thereby leaving an environment in which bacteria could persist. Therefore, to more accurately assess the caries-preventive effect of disinfectants alone, further experiments should evaluate their ability to inhibit bacterial growth within the residual biofilms.

In conclusion, this study suggests that *S. mutans*, a cariogenic bacterium, is less susceptible to bactericidal action than non-cariogenic bacteria such as *S. mitis, S. sanguinis, S. oralis, S. salivarius*, and *S. gordonii.* Among the three disinfectants tested, ozone water produced the lowest ATP values for *S. mutans,* and both the ATP measurements and LIVE/DEAD biofilm staining revealed no significant bactericidal effects on non-mutans streptococci. These results suggest that using 4–5 mg/L ozone water as a mouthwash could suppress caries while preserving the oral microbiota.

## CRediT authorship contribution statement

**M. Komatsubara:** Formal analysis, Data curation. **A. Watanabe:** Investigation, Funding acquisition. **S. Kuwagi:** Formal analysis, Data curation. **J. Uchiyama:** Writing – original draft. **K. Gotou:** Writing – original draft. **K. Yokota:** Writing – original draft, Supervision.

## Ethics statement

This study did not involve human participants, personal data, or animals, and therefore did not require approval from an ethics committee. All procedures were conducted in accordance with relevant guidelines and regulations.

## Funding sources

This study was supported by a Grant-in-Aid for Scientific Research from the 10.13039/501100001691Japan Society for the Promotion of Science (JSPS) (KAKENHI, Grant Number 22K10290).

## Conflict of interest statement

The authors declare no conflicts of interest.
